# Evaluation of geospatial methods to generate subnational HIV prevalence estimates for local level planning

**DOI:** 10.1097/QAD.0000000000001075

**Published:** 2016-05-11

**Authors:** The Subnational Estimates Working Group of the HIV Modelling Consortium

**Affiliations:** Department of Infectious Disease Epidemiology, Imperial College London, London, UK.

**Keywords:** health planning/organization and administration, health policy, HIV infections/epidemiology, HIV seroprevalence, HIV/infections prevention and control, population surveillance/methods

## Abstract

**Objective:**

There is evidence of substantial subnational variation in the HIV epidemic. However, robust spatial HIV data are often only available at high levels of geographic aggregation and not at the finer resolution needed for decision making. Therefore, spatial analysis methods that leverage available data to provide local estimates of HIV prevalence may be useful. Such methods exist but have not been formally compared when applied to HIV.

**Design/methods:**

Six candidate methods – including those used by the Joint United Nations Programme on HIV/AIDS to generate maps and a Bayesian geostatistical approach applied to other diseases – were used to generate maps and subnational estimates of HIV prevalence across three countries using cluster level data from household surveys. Two approaches were used to assess the accuracy of predictions: internal validation, whereby a proportion of input data is held back (test dataset) to challenge predictions; and comparison with location-specific data from household surveys in earlier years.

**Results:**

Each of the methods can generate usefully accurate predictions of prevalence at unsampled locations, with the magnitude of the error in predictions similar across approaches. However, the Bayesian geostatistical approach consistently gave marginally the strongest statistical performance across countries and validation procedures.

**Conclusions:**

Available methods may be able to furnish estimates of HIV prevalence at finer spatial scales than the data currently allow. The subnational variation revealed can be integrated into planning to ensure responsiveness to the spatial features of the epidemic. The Bayesian geostatistical approach is a promising strategy for integrating HIV data to generate robust local estimates.

## Abstract 2

Supplemental Digital Content is available in the text

## Introduction

Historically, country epidemics have been considered as fairly homogenous and been broadly classified as ‘generalized’ or ‘concentrated’ [1]. However, such an approach fails to capture the often substantial local level variation in the patterns of risk and transmission, key drivers of the epidemic, and availability of services observed [2]. Indeed, 13 of 33 countries in sub-Saharan Africa report at least five-fold differences in adult prevalence between provinces [3].

This spatial heterogeneity has profound implications for all aspects of monitoring the epidemic and planning the response. There have been a large number of examples of ways in which spatial data can be used to improve HIV planning, including identification of places or populations at highest risk [4], allocation of resources across locations [5], in understanding local level changes and monitoring the epidemic [6], in interpreting gaps in service provision [7], in understanding reasons for different biases in available surveillance data [8], and in tailoring services and local level targeting of intervention [9].

Although it is critical that planners have an understanding of local level variations in the intensity of the HIV epidemic, practical and financial constraints that restrict the size of surveys and surveillance systems commonly used to monitor generalized epidemics may inhibit this. Robust estimates of subnational HIV prevalence are typically available at the first administrative level (often termed ‘provinces’), but not at more local levels which may be needed for programme planning (such as the district or county levels).

There are several candidate methods that could be used to generate local subnational estimates of HIV prevalence (Table 1). These include the method that the Joint United Nations Programme on HIV/AIDS (UNAIDS) has used to generate maps of high-burden countries [23] and a method which has been used to estimate malaria prevalence patterns [14,15]. However, the performance of these methods has not been evaluated or compared in the context of HIV epidemiology. Here, we conduct a formal evaluation and comparison of subnational HIV prevalence estimates generated by these candidate methods.

## Methods

Six candidate methods were included in this study (labelled models 1–6 in Table 1). Key characteristics are described in Table 1.

Some mapping strategies use sampled HIV prevalence data from household surveys only and do not use ancillary spatial information (e.g. road network or vegetation coverage). PrevR (model 1) is such a method [10], and it has been recommended by UNAIDS on the basis of it being straightforward to implement and available for immediate use.

This approach is compared with methods that do leverage information on other features (‘covariates’) which can enhance predictions of HIV prevalence (data sources are described in Table S1). Such data are particularly useful if they are sampled at a greater geographical resolution than the HIV prevalence data. Models 2–6 fall into this category. Among these, there are important differences in the theoretical framework behind them, which influence their ability to deal with uncertainty and the computational load (Table 1).

Finally, some of these methods estimate a continuous ‘surface’ of HIV prevalence (models 1–3), whereas others instead aim to provide predictions for the aggregate level, namely the subnational units under consideration directly, for example, a district level prevalence estimate (models 4–6).

Two different validation procedures were used to assess the performance of the methods, depending on whether the method produces a ‘surface’ of prevalence or can give estimates at aggregate subnational level. Data from three countries (Tanzania, Kenya, Malawi) with generalized epidemics were used. These countries were chosen as they encompass variation in epidemic patterns and data availability.

### Internal validation

The performance of those models that produce continuous HIV prevalence surfaces (models 1–3) was assessed using internal validation. A proportion of observed data is held back as a ‘test’ dataset, before the method is used. Then, the test data are used to challenge the model prediction at sample locations. This test dataset was either a single point, namely a demographic and health survey (DHS) cluster [leave out one cross validation (LOOCV), (models 1 and 3)] or a larger proportion of the data [partitioned data hold back (PDHD), (models 1 and 2)]. Which strategy was applied (i.e. LOOCV or PDHD) for a given spatial method was dependent on the computational intensity of the mapping approach.

The resulting root mean squared error (RMSE) (Eq. 1) between prediction and data was calculated. The RMSE estimates are in the same units as the surface (i.e. prevalence) and the lower the RMSE, the closer the model prediction is to the observed data.
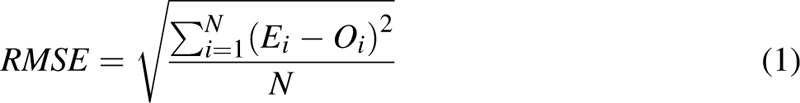


where *E* is the predicted values, *O* the observed values, *N* the total number of locations, and *i* each omitted location.

### External validation (cross-year comparison of estimates)

External validation was also conducted through comparing mapped predictions with data from an earlier survey year. This approach takes advantage of the location of clusters often being different in different survey years in a country, and assumes that the true spatial pattern of HIV prevalence is conserved over time. All three countries considered have surveys for more than one DHS round (Malawi: 2004 and 2010, Tanzania: 2007/2008 and 2012, Kenya: 2003 and 2008/2009) allowing for such an analysis. For methods that produce a continuous surface (models 1–3), prevalence surfaces were produced using only the data from the later survey. Then, a comparison was made between the observed values for each cluster in the earlier survey year and the predicted prevalence in the corresponding location in the year of the later survey.

The approach was repeated for all methods at the level of the first administrative unit for Malawi (district level), as not all methods produce continuous prevalence surfaces. For those methods which produced predicted surfaces (models 1–3), the average of the values of the surface within each district boundary was calculated, to allow for comparison with those methods which produce district level estimates directly (models 4–6). The accuracy of district level predictions in comparison to the data from the earlier survey year was summarized by the RMSE.

## Results

**Fig. 1:**
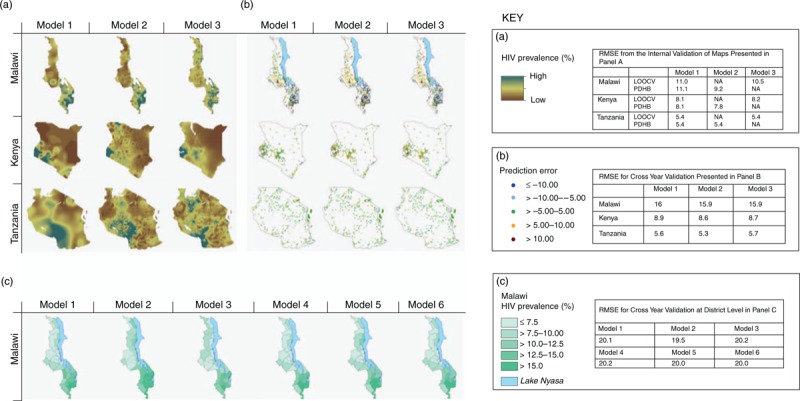
Results from the validation exercises.

Figure 1a presents the continuous prevalence surfaces for each country using models 1–3. Very substantial within-country variations in HIV prevalence are revealed by all methods. In particular, methods indicate a prevalence gradient from east to west in Kenya, south to north in Malawi, and a focus of high prevalence in south-west Tanzania.

Panel (a) gives the HIV prevalence surfaces from models 1–3, and the corresponding Root Mean Squared Error (RMSE) values from the internal validation [either Leave Out One Cross Validation (LOOCV) or Partitioned Data Hold Back (PDHD)] are given in the tables in the Key. Panel (b) gives the error maps from the external validation procedure using the cross year comparison of spatial patterns and the corresponding RMSE values for models 1–3. Panel (c) gives the estimates of HIV prevalence for each district in Malawi and the corresponding RMSE value for all models (1–6). Note that the results presented in panel (a) and panel (b) are only for models 1–3 as they are only evaluating those methods which produce continuous prevalence surfaces.

There is substantial variation in the degree of local level variation suggested by the models. Methods that bring in additional data tend to produce estimates with a more complex spatial structure, which is related to road networks, among other factors, although uncertainty in the estimates about this is great (not shown). In contrast, the PrevR approach gives a smooth surface.

The internal validation procedure suggests that all methods can produce estimates of HIV prevalence at unsampled locations with a similar, and reasonable, level of accuracy (RMSE values: Fig. 1a). Among the methods, the Bayesian geostatistical approach (model 2), gives marginally the best RMSE values consistently across all the countries (Fig. 1: RMSE values displayed in the key for each panel).

The external validation for the continuous surfaces shows that the methods typically are successful in predicting prevalence in unsampled locations (Fig. 1b). The greatest difference in prediction error is between countries rather than between methods, and all methods have similar spatial pattern in the errors. Among models 1–3 (those that could do this test), model 2 gives the lowest RMSE across all countries. All methods give higher RMSE values than in the internal validation exercise described above as they are being used to predict the spatial distribution from a different year and the epidemic will have changed.

The external validation for the district level in Malawi (Fig. 1c), shows that whereas all the methods give the same broad spatial trends, differences in some districts are quite pronounced and overall errors are much greater. As prevalence may vary widely within an administrative region, particularly between urban and rural areas, an averaged value for each administrative region gives wider differences than the point by point comparison. Again, model 2 gives the lowest RMSE, and gives the most accurate prediction.

## Discussion

Generating subnational estimates of HIV prevalence will be crucial to informing a locally tailored response to the HIV epidemic. This analysis has provided a number of key insights as to how countries can best utilise available spatial data.

First, the magnitude of the error and accuracy of predictions appears to depend most on the prevalence level in the country of interest and the characteristics of the survey sample, rather than the estimation method used. Because of this, we see a greater difference in the accuracy of predictions between countries rather than between methods. For this reason, some confidence in predictions from these spatial methodologies comes from their relative consistency in performance across all validation procedures described. The method used already by UNAIDS (model 1: PrevR) performs similarly to most other methods and so greater confidence can be afforded in the results. As a result of these analyses, we recommend that Bayesian geostatistical approach (model 2) be developed further, as the performance of this method was consistently the strongest. This method has been applied extensively to other infections [13], and has many desirable characteristics, in particular a formal accounting of uncertainty and the explicit leveraging of other geographic data.

Second, the methods appear to work reasonably well, and can capture the broad spatial trends in prevalence observed across countries. Arguably, in these high prevalence settings, these methods would usefully distinguish areas of very high prevalence from those with very low prevalence.

The mapping methods described can be further developed, particularly through integration of different data sources alongside the DHS, in particular, antiretroviral therapy and prevention of mother-to-child transmission programme data, antenatal clinic surveillance, and, in the future, case-reporting data. Doing so would require building upon earlier work [8] to assess how different data sources may feed into prevalence mapping in a manner that reflects the different biases, underlying populations and spatial coverage of these data. Furthermore, although the tools outlined can fill critical gaps at this time, they do not mitigate the need for future additional local data collection and reporting to strengthen more localized responses to the HIV epidemic.

National epidemics cannot continue to be assessed as a whole when there is clear evidence of substantial subnational heterogeneity. Subnational indicators should be integrated into all national planning, monitoring, and evaluating processes performed routinely. Existing tools, such as Spectrum/Estimation and Projection Package modelling software, are already being adapted to explicitly examine the epidemic in subnational areas [24]. The increasing availability of georeferenced data and mapping tools provides us with the opportunity to be responsive to the subnational features of HIV epidemics to improve intervention planning.

## Acknowledgements

Wrote the first draft of the article: T.B.H., S.-J.A. Contributed to data collation and analysis: T.B.H., S.-J.A., D.F., C.R.B., N.J.H., D.F.C., I.M., J.L., N.B.K., P.W.G., S.B., S.O.M.M., A.S and L.Z. Contributed to the writing of the article: all authors.

Funding: This study was funded by the Bill & Melinda Gates Foundation through a grant for the HIV Modelling Consortium (www.hivmodelling.org) to Imperial College London. The funders had no role in study design, data collection and analysis, decision to publish, or preparation of the manuscript.

The findings and conclusions in this report are those of the authors and do not necessarily represent the official position of the US Centers for Disease Control and Prevention (CDC).

The Subnational Estimates Working Group of the HIV Modelling Consortium is as follows:

Chair: Timothy B. Hallett (Department of Infectious Disease Epidemiology, Imperial College London, London, UK), Members: Sarah-Jane Anderson (Department of Infectious Disease Epidemiology, Imperial College London, London, UK), Cynthia Adobea Asante (Ghana AIDS Commission, Accra, Ghana), Noah Bartlett (USAID, Washington, DC, USA), Victoria Bendaud (UNAIDS, Geneva, Switzerland), Samir Bhatt (University of Oxford, Oxford, UK), Clara R. Burgert (ICF International, The Demographic and Health Surveillance Program, Rockville, MD, USA), Diego Fernando Cuadros (Weill Cornell Medical College in Qatar, Cornell University, Qatar Foundation - Education City, Doha, Qatar), Janet Dzangare (AIDS & TB Unit, Ministry of Health and Child Care, Harare, Zimbabwe), Daniela Fecht (Small Area Health Statistics Unit, Imperial College London, London, UK), Peter William Gething (University of Oxford, Oxford, UK), Peter D. Ghys (UNAIDS, Geneva, Switzerland), James M. Guwani (UNAIDS, Johannesburg, South Africa), Nathan Joseph Heard (US Department of State, Washington, DC, USA), Ezekiel Kalipeni (University of Illinois at Urbana-Champaign, Champaign, IL, USA), Ngianga-Bakwin Kandala (Department of Mathematics and Information Sciences, Northumbria University, Newcastle upon Tyne, UK), Andrea A. Kim (US Centers for Disease Control and Prevention, Atlanta, USA), Isaiah Doe Kwao (Ghana AIDS Commission, Accra, Ghana), Joseph Larmarange (CePeD, IRD, Paris, France), Samuel O. M. Manda (Biostatistics Research Unit, South African Medical Research Council, Pretoria, South Africa and School of Mathematics, Statistics and Computer Science, University of KwaZulu-Natal, Pietermaritzburg, South Africa), Imelda K. Moise (University of Miami, Miami, FL, USA), Livia S. Montana (Harvard University Center for Population and Development Studies, Cambridge, MA, USA), Daniel N. Mwai (The Palladium Group, University of Nairobi, and USAID Health Policy Project, Nairobi, Kenya), Samuel Mwalili (US Centers for Disease Control and Prevention, Nairobi, Kenya), Ashton Shortridge (Department of Geography, Michigan State University, MI, United States), Frank Tanser (Africa Centre for Health and Population Studies, University of KwaZulu-Natal, Mtubatuba, South Africa, School of Nursing and Public Health, University of KwaZulu-Natal, Durban, South Africa), Ian Wanyeki (Futures Group, Nairobi, Kenya) and Leo Zulu (Department of Geography, Michigan State University, MI, United States).

The prevR package was developed with financial support of ANRS (Agence Nationale de Recherche sur la Sida et les hépatites virales) and IRD (Instituit de Recherche pour le Développement).

### Conflicts of interest

S.J.A. received personal fees from the Bill & Melinda Gates Foundation and Anansi Health outside of the submitted work. C.B. was supported by US Agency for International Development (USAID) with support for travel from USAID and UNAIDS. D.F. reports consultant fees from Imperial College Consultants. P.W.G. is a Career Development Fellow (#K00669X) jointly funded by the UK Medical Research Council (MRC) and the UK Department for International Development (DFID) under the MRC/DFID Concordat agreement and receives support from the Bill and Melinda Gates Foundation (#OPP1068048). These grants also support S.B. T.B.H. received grants and personal fees from the Bill & Melinda Gates Foundation during the conduct of the study; grants and personal fees (prior to the conduct of the work) from World Bank; grants from UNAIDS, and The Rush Foundation; personal fees from the University of Washington, New York University, and Global Fund outside of the submitted work. J.L. is supported by IRD, and reports personal fees from UNAIDS and grants from ANRS.

## Figures and Tables

**Table 1:** Key characteristics of the candidate spatial methods.

Methods						
Method name	Summary of method	References	Use of covariates	Estimation of uncertainty	Ease of use	Unit of analysis
Model 1: kernel density estimation with adaptive bandwidth (prevR)	The numbers of individuals tested and found positive at each DHS cluster location are plotted, and smoothed intensity surfaces are generated by computing average infection rates within a moving window (a ’kernel’). Creation of the smoothed surface relies on the specification of the kernel at each cluster; that are combined to make the intensity surface. The prevalence surface is the ratio of the intensity surface of the number who tested positive to the surface of the total number tested	Larmarange *et al.*, 2011 [10]; Davies and Hazelton, 2010 [11]	Does not use information on covariates	Able to provide an indication of how much data were used in producing the estimate	Straightforward- well documented, open source	Pixel level-provides estimates for small areas to give a continuous surface
Model 2: model-based geostatistics	Bayesian, or ’model-based’ geostatistical approaches are a special class of generalized linear mixed models. They extend classical geostatistical methods such as kriging to allow, among other features, formal incorporation of: sampling error in the observed data; relationships with covariates (and the uncertainty in the form of these relationships); uncertainty in the spatial autocorrelation structure of the outcome variable. The model can be fit using computational methods like MCMC	Diggle *et al.*, 1998 [12]; Patil *et al.*, 2011 [13]; Gething *et al.*, 2011 [14]; Gething *et al.*, 2012 [15]	Uses information on covariates	Conducted within a Bayesian framework, and is able to provide a rigorous assessment of the uncertainty of the estimated value at each location on the mapped surface	Complex but could be adapted for the end user	Pixel level- provides estimates for small areas to give a continuous surface
Model 3: kriging of covariates with logistic regression	This method involves two steps, the prediction of the spatial distribution of each covariate independently using kriging and combination of these layers using a nonspatial logistic regression model to generate HIV prevalence predictions	Cuadros, 2014 [16]; Klienschmidt *et al.*, 2000 [17]	Uses information on covariates	Does not provide estimates of uncertainty	Moderately complex involves multiple steps that require expertise	Pixel level- provides estimates for small areas to give a continuous surface
Model 4: shared spatial component	The shared spatial component model was originally developed to map different diseases likely to have similar spatial distributions because of shared risk factors. The model has both shared components and components specific to each disease of interest. The shared spatial component model was applied here to incorporate information from both the DHS and ANC data sources. This model looks at discrete areas (administrative units) and is constructed within a Bayesian framework	Manda *et al.*, 2009 [18]; Knorr-Held *et al.*, 2001 [19]	Uses information on covariates	Able to provide estimates of uncertainty for the unit of analysis (administrative region)	Moderately complex involves multiple steps that require expertise	Provides estimates for the administrative unit
Model 5: spatio-statistical aggregate method: regression at an aggregated scale (administrative unit)	Following an exploration of regression kriging at the cluster level for Malawi, it was found that the spatial predictive power of the model was limited because of weak spatial structure. As a result an aggregate scale was explored instead. This method involves aggregation (through simple averaging) of the DHS clusters to administrative regions, followed by OLS multiple regression to examine the relationship between selected covariates and HIV prevalence. The administrative unit could be the target administrative unit, or it may be that the optimal unit for predictive power is lower	Moise *et al.*, 2014 [20]; Moise *et al.*, 2014 [21]	Uses information on covariates	Does not provide estimates of uncertainty	Moderately complex involves multiple steps that require expertise	Provides estimates for the administrative unit
Model 6: Bayesian geo-addative mixed model	This approach is similar to model 2 in that it is a Bayesian approach that is able to incorporate the sampling error, relationships with covariates, and uncertainty in the spatial autocorrelation structure. This model differs from model 2 in that it looks at the patterns of prevalence across discrete areas (administrative units) rather than continuous space as in model 2, and accounts for the spatial dependencies between these neighbouring areas. This model uses computational methods (MCMC) for inference and checking of the model. It is conducted using BayesX software.	Kandala *et al.*, 2011 [22]	Uses information on covariates	Able to provide estimates of uncertainty for the unit of analysis (administrative unit)	Moderately complex involves multiple steps that require expertise	Provides estimates for the administrative unit

ANC, antenatal clinic; DHS, demographic and health survey; MCMC, Markov chain Monte Carlo; OLS, ordinary least squares.
